# Cortical Processing of Swallowing in ALS Patients with Progressive Dysphagia – A Magnetoencephalographic Study

**DOI:** 10.1371/journal.pone.0019987

**Published:** 2011-05-20

**Authors:** Inga K. Teismann, Tobias Warnecke, Sonja Suntrup, Olaf Steinsträter, Linda Kronenberg, E. Bernd Ringelstein, Reinhard Dengler, Susanne Petri, Christo Pantev, Rainer Dziewas

**Affiliations:** 1 Department of Neurology, University of Muenster, Muenster, Germany; 2 Institute for Biomagnetism and Biosignalanalysis, University of Muenster, Muenster, Germany; 3 Department of Neurology, Hannover Medical School, Hannover, Germany; The University of Western Ontario, Canada

## Abstract

Amyotrophic lateral sclerosis (ALS) is a rare disease causing degeneration of the upper and lower motor neuron. Involvement of the bulbar motor neurons often results in fast progressive dysphagia. While cortical compensation of dysphagia has been previously shown in stroke patients, this topic has not been addressed in patients suffering from ALS. In the present study, we investigated cortical activation during deglutition in two groups of ALS patients with either moderate or severe dysphagia. Whole-head MEG was employed on fourteen patients with sporadic ALS using a self-paced swallowing paradigm. Data were analyzed by means of time-frequency analysis and synthetic aperture magnetometry (SAM). Group analysis of individual SAM data was performed using a permutation test. We found a reduction of cortical swallowing related activation in ALS patients compared to healthy controls. Additionally a disease-related shift of hemispheric lateralization was observed. While healthy subjects showed bilateral cortical activation, the right sensorimotor cortex was predominantly involved in ALS patients. Both effects were even stronger in the group of patients with severe dysphagia. Our results suggest that bilateral degeneration of the upper motor neuron in the primary motor areas also impairs further adjusted motor areas, which leads to a strong reduction of ‘swallowing related’ cortical activation. While both hemispheres are affected by the degeneration a relatively stronger activation is seen in the right hemisphere. This right hemispheric lateralization of volitional swallowing observed in this study may be the only sign of cortical plasticity in dysphagic ALS patients. It may demonstrate compensational mechanisms in the right hemisphere which is known to predominantly coordinate the pharyngeal phase of deglutition. These results add new aspects to our understanding of the pathophysiology of dysphagia in ALS patients and beyond. The compensational mechanisms observed could be relevant for future research in swallowing therapies.

## Introduction

Amyotrophic lateral sclerosis is a progressive degenerative motor neuron disease affecting the upper as well as the lower motor neuron [1]. Dysphagia is one of the most important clinical problems encountered in ALS. It appears typically several months after the onset of the disease. However, about 25% of patients initially exhibit bulbar symptoms, where bulbar and pseudobulbar palsy are present. Early swallowing-related problems in ALS patients mainly occur in the pharyngeal stage of deglutition including prolonged muscle activity of the laryngeal elevators and a delayed opening of the upper oesophageal sphincter [2], [3].

Magnetoencephalography (MEG) can monitor cortical activity with a high temporal and spatial resolution [4]. Motor tasks have been shown to result in event-related desynchronisations (ERD) of the cortical beta rhythm in cortical motor areas [5], [6]. In the last few years synthetic aperture magnetometry (SAM) based on whole-head MEG has been demonstrated to be a reliable method to examine the complex function of swallowing in humans [7], [8], [9], [10], [11], [12], [13], [14]. While the artifacts caused by oropharyngeal muscle activation during the act of swallowing make it difficult to study activation in subcortical and bulbar structures, the cortical areas especially the sensorimotor areas can be examined in detail.

In healthy subjects several functional brain imaging studies have examined the cortical activation of human swallowing. Among other brain areas a bilateral activation of the primary and secondary sensorimotor cortical areas was found consistently (Brodmann Areas [BA] 1–6). Up to now only few studies focussed on the cortical swallowing processing in dysphagic patients. In particular, the progressive dysphagia due to motor impairment in ALS patients has not been explored by functional imaging of the human brain. In a recent study of our group we performed the swallowing paradigm on patients suffering from Kennedy Disease (KD). Similar to dysphagia in ALS, these patients also demonstrate with mainly pharyngeal phase dysphagia. Here we found an increase in bilateral sensorimotor activation with a right hemispheric lateralization [9]. The predominantly active right hemisphere can be explained by the hypothesis of a task sharing for different components of deglutition between the two hemispheres. The left hemisphere more selectively mediates the oral phase and therefore volitional components, whereas the right hemisphere contributes more to the pharyngeal phase and automatic reflexive aspects of swallowing. First insights into this topic were generated by lesion studies [15] and could be supported by a previous study on healthy subjects by our group [10]. The right hemispheric lateralization observed in Kennedy patients therefore indicates cortical compensation of pharyngeal phase dysphagia.

In the present study we employed whole-head MEG and SAM analyses to study cortical activity during self-paced volitional swallowing on fourteen patients suffering from sporadic ALS with bulbar onset. We hypothesized a decrease in cortical activation compared to healthy controls due to the degeneration of the motor neurons. Analogous to the effects observed in patients with Kennedy disease we expected lateralization of cortical activation to the right hemisphere.

## Results

### FEES examination

Flexible endoscopic evaluation of swallowing (FEES) revealed signs of mild to severe dysphagia in all 14 patients. All patients showed residues in the valleculae and pyriforme sinus, weakness of pharyngeal constriction and reduced laryngeal elevation with the mildly dysphagic patients (MDG) being less impaired than severely dysphagic patients (SDG). Impairment of bolus preparation and food transport was found mainly in SDG patients. All patients swallowed small amounts of water without penetration or aspiration. Soft touch of the pharyngeal wall with the endoscope was percepted by all patients in both groups.

### Swallowing Screening Test

The ANOVA and *post-hoc* t-tests applied to the swallowing screening-test results revealed a significant weaker swallowing ability of SDG patients compared to controls as well as to MDG patients in all 4 measured items. Comparing the MDG patients to controls, worse results were found in the patient group in all measured items except for the volume per swallow (p  =  0.113; see table 1).

**Table 1:** Results of the Swallowing Screening test for control group, mildly dysphagic group (MDG) and severely dysphagic group (SDG).

	Controls			MDG		SDG
**Volume per Swallow (ml)**	18.6 +/− 3.2		§**	14.1 +/− 3.2	$**	6.6 +/− 2.1
**Duration per Swallow (s)**	1.2 +/− 0.4	&*	§**	2.4 +/− 1.1	$*	4.5 +/− 1.8
**Capacity (ml/s)**	17.7 +/− 6.0	&*	§***	7.1 +/− 3.4	$*	1.7 +/− 1.1

Mean and standard deviation for age and all three measured items are shown. Significant differences between the three groups are specified by Bonferroni corrected t-tests.

controls vs. MDG (&); controls vs. SDG (§); MDG vs. SDG ($).

p < 0.05;

**p < 0.01;

***p < 0.001

### Behavioral data during MEG recordings

Compared to the swallowing screening test swallows during MEG recordings were less demanding. The infusion rate was chosen to make swallows comfortable possible for control subjects as well as for dysphagic patients. Additionally the time between two swallows could be chosen by each subject. Therefore all participants tolerated the MEG examination without any difficulties. Number of swallows, total swallowing time and duration per swallow during EMG recording did not differ between the three groups (p > 0.05) (see table 2).

**Table 2:** Results of the EMG recording during 15 min of MEG measurement.

	No. of Swallows	Duration per Swallow (s)	total swallowing time (s)
**Severe dysphagic group (SDG)**			
A1	70	1.7	119.25
A2	68	3.53	240.04
A3	26	2.23	57.93
A4	52	1.51	78.54
A5	91	1.66	151.39
A6	61	4.62	281.91
A7	30	1.42	42.55
mean	56.86	2.38	138.80
**Mild dysphagic group (MDG)**			
B1	82	1.84	151.21
B2	31	3.53	109.34
B3	35	2.33	83.84
B4	56	3.86	216.32
B5	38	2.54	96.63
B6	56	4.45	249.29
B7	61	2.65	161.59
mean	51.29	3.03	152.60
**Healthy controls**			
C1	66	4.86	321,01
C2	71	4.2	298.54
C3	44	1.85	81.19
C4	53	1.54	81.68
C5	83	2.07	171.76
C6	49	1.63	79.69
C7	42	4.8	201.56
mean	58.29	2.99	130.63

The RMS (root mean square) of the EMG amplitude (EMG power) across the complete swallowing execution interval (M0 – M2) revealed a significant main effect of GROUP (F_(2, 6)_  =  4.279, p < 0.05). Post-hoc analyses showed that EMG power was significantly larger in the SDG compared to the control group (p < 0.05), while difference between the MDG and the controls did not reach significance (p  =  0.201).

### Individual SAM results

In all seven healthy control subjects individual SAM analysis of the swallowing execution phase resulted in ERD of the beta and low gamma frequency band bilateral in the sensorimotor cortex (see table 3). In 6 of these subjects additional sensorimotor ERD was found in the alpha frequency range. A trend for left hemispheric lateralization of sensorimotor beta activation was found in this group without reaching significance (p < 0.1).

**Table 3:** MNI coordinates and Brodmann areas of the maximum beta synchronization of both hemispheres in each individual subject and patient.

	left hemisphere				right hemisphere			
	X	Y	z	Brodmann	x	y	z	Brodmann
**Severe dysphagic group**								
A1	−21	−24	48	4	38	−26	62	4
A2	−48	−24	54	1	57	−18	48	1
A3	−48	3	45	6	54	21	15	45
A4	−63	−39	45	40	64	−39	51	40
A5	−60	−15	39	4	45	−26	65	3
A6	−45	−33	48	40	45	−30	60	2
A7	−6	−33	75	6	42	−12	60	4
**Mild dysphagic group**								
B1	−33	−33	60	4	36	−57	48	40
B2	−27	−42	54	3	60	27	24	45
B3	−31	−22	64	4	3	−42	63	5
B4	−42	−30	66	1	42	-9	60	6
B5	−39	−33	57	3	16	15	54	6
B6	−21	−21	63	6	36	−15	57	4
B7	−36	−24	45	3	51	−27	36	2
**Controls**								
C1	−36	−24	75	6	9	−39	72	4
C2	−51	−3	60	6	30	−15	75	6
C3	−36	−21	72	6	15	−27	72	4
C4	−6	−24	75	6	6	−12	72	6
C5	−57	−9	24	4	36	−21	54	4
C6	−33	−36	54	3	27	−21	60	6
C7	−21	−24	60	4	33	−6	54	6

In all 7 patients of the MDG beta ERD of the sensorimotor cortex was found, while gamma ERD of these areas was only found in 3 and alpha ERD in 5 patients. 6 of the 7 patients with MDG showed clear right hemispheric lateralization of the ERD in the beta frequency range (p < 0.05).

In the SDG all 7 patients showed ERD of the beta band during swallowing execution. Gamma desynchronization of the sensorimotor cortex was found in 4 and alpha desynchronization in 2 of these patients. Again, 6 of these patients demonstrated right hemispheric lateralization in the beta ERD (p < 0.05).

In all three examined frequency bands no systematic brain activation was observed in other cortical areas in any of the three groups.

### Group SAM results

**Figure 1:**
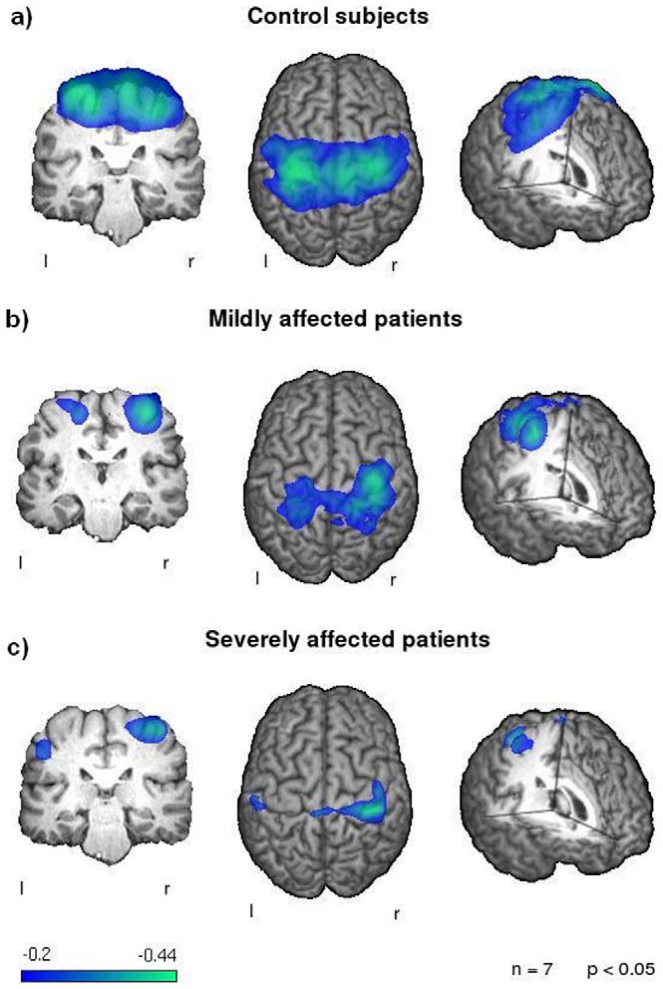
Event related desynchronization. Changes in the beta-frequency-band during swallowing execution compared to the resting stage. The color bar represents the t-value. a) Control subjects show a strong and bilateral activation of the primary sensorimotor cortex. b) In the MDG weaker activation of both sensorimotor cortices can be found. The right hemispheric activation is stronger compared to the left side. c) In the SDG only little activation of the left hemisphere is observable. The right hemispheric activation is reduced compared to healthy controls and to the MDG. The maximum of activation is located more posterior compared to the control group, corresponding to the postcentral gyrus.

Group analysis of SAM results showed significant ERDs in the alpha, beta and low gamma frequency bands (p < 0.05) of the primary sensorimotor cortex during the execution phase in the control group. The strongest and broadest activation was found in the beta band (see figure 1a). ERD were located bilaterally around the central sulcus involving the pre- and postcentral gyrus with a slight but not significant left hemispheric lateralization. Group analysis showed significant bilateral beta ERD located in the primary and secondary sensorimotor areas including BA 1, 2, 3, 4, 5, 6, 7 and 40 (p < 0.05) with a maximum of activation in BA 4 in both hemispheres. Analysis of low gamma and alpha activation revealed a similar but weaker activation of the sensorimotor cortex. Noticeable is a distinct right hemispheric dominance of the low gamma ERD.

In both patient groups significant beta ERDs of the primary sensorimotor cortex were found (BA 1,2,3 and 4). As in the group of control subjects the maximum of activation was located in BA 4 in both groups of patients and in both hemispheres (see figure 1b and c). Gamma and alpha desynchronizations in both patient groups were located bilaterally around the postcentral gyrus without reaching significance.

Compared to the control group the maximum pseudo-t value in the left hemisphere decreased in the MDG to 68% and even further to 60% in the SDG. This effect was less strong in the right hemisphere, where the maximum pseudo-t value of the MDG is still 97% compared to the control group and 77% in the SDG. In both patients groups a right hemispheric lateralization was found. The effect was stronger in the SDG (LI: −.702) compared to the MDG (LI: −.567).

### Group comparison

Group comparison of all three groups revealed significantly stronger cortical activation of the bilateral primary sensorimotor cortex in the control group compared to both groups of ALS patients (p < 0.05). Compared to the control group clear right hemispheric lateralization was observed in both patient groups. Comparison of both ALS groups showed a similar pattern of beta ERD with a significantly stronger and broader activated area in the MDG compared to the SDG group (p < 0.05).

## Discussion

This study is one of the first to investigate the effect of motor neuron degeneration in ALS on the cortical processing of human swallowing. We found significant reduction of cortical sensorimotor activation in ALS patients compared to healthy controls. As shown by the comparison of mildly dysphagic and severely dysphagic ALS patients, this effect increases with progression of the disease.

### Localization of cortical activation

Cortical ERD in all three examined groups were observed in the bilateral superior sensorimotor areas. These activated cortical areas are located more superior and medial with respect to what has to be expected based on the homunculus. Additionally the swallowing related cortical activation is spread extensively in primary and secondary motor and sensory areas in both hemispheres, which is mainly observed in healthy controls. This is a well known phenomenon in functional brain imaging of human swallowing processing. It has been observed in several former MEG studies by our group [7], [9], [10], [11], [12], [13], [14]. But also other studies using TMS, PET and MEG led to similar patterns of activation [8], [16], [17]. As reason for this widespread cortical activation in swallowing processing projections to and from the swallowing tract located in rostral non-primary motor areas are suggested by Hamdy and co-workers [17]. The swallowing network therefore exceeds the primary sensorimotor cortex and involves secondary areas.

### Potential reasons for the reduction of cortical activation in ALS

The present data demonstrates a reduction in the sensorimotor activation in dysphagic ALS patients. While simple motor tasks like finger flexion or button press result in an increase of cortical activation [18], [19], [20], [21], [22], bulbar activation has shown to cause a decrease in the corresponding brain activity. A recent pilot fMRI study on 5 dysphagic ALS patients by Li and co-workers found a decrease of swallowing related cortical activation in the primary sensorimotor system in dysphagic ALS patients but not in non dysphagic patients or in healthy controls [23]. A fMRI study examined vertical tongue movements in ALS patients with bulbar involvement [24]. These patients showed a significant decrease of cortical and thalamic activation, while limb movements resulted in an increase of cortical activity. Until now it is unknown why in ALS patients limb movements result in an increase of cortical activation while swallowing and tongue movements cause a reduction.

Interestingly, cortical swallowing activity, though lower in levels of activation in ALS patients, is focused on the central area of the primary motor cortex without involving more mesial areas or the secondary sensorimotor cortex. This might reflect a disturbance of the swallowing network including the secondary and tertiary pericentral areas of both hemispheres, leading to a strongly reduced activation in these areas. Additionally, bilateral involvement of the primary motor cortex in deglutition may be crucial for the lack of motor system compensation. It is also conceivable that adaptive changes in subcortical structures, in particular the brainstem, might have been missed in the present study due to technical limitations of MEG.

The question arises whether the reduction of activation is predominantly caused by degeneration of the upper or the lower motor neuron. Degeneration of the upper motor neuron leads to morphologic [25], [26] as well as functional changes in the primary motor cortex. In patients with primary lateral sclerosis, which mainly affects the upper motor neuron [27], a decreased thickness of the primary motor cortex was found [28]. PET studies examining motor tasks on ALS patients resulted in significantly lower cerebral blood flow in the primary sensorimotor cortex compared to healthy controls [29] and patients with disorders confined to the lower motor neuron [20].

In contrast to these results an isolated degeneration of the lower motor neuron is associated with compensatory increase of the related cortical activation. Structural changes were observed in different cortical motor areas and the corticospinal tract in ALS but not in healthy subjects or in patients suffering from progressive muscular atrophy (PMA), where only the lower motor neuron is affected [30], [31]. In a recent study of our group we performed the present swallowing paradigm on patients suffering from Kennedy Disease (KD). Here we found an increase in bilateral sensorimotor activation with a right hemispheric lateralization [9]. We therefore suppose that the reduction of the bilateral cortical ERD during swallowing execution can be mainly explained by the degeneration of the upper motor neuron.

This might also lead to compensational mechanisms in the brainstem. Until about 10 years ago swallowing was thought to be coordinated only by the brainstem. New functional brain imaging methods proved the influence of several cortical areas on deglutition [7], [16], [32]. We suppose that these findings can be explained as follows: ALS causes severe disturbance of motor control of the swallowing muscles. As a consequence, the central pattern generators in the brainstem possibly have to take over part in swallowing coordination. The increased EMG-power present in ALS patients may therefore reflect a less well-coordinated act of swallowing. Unfortunately, the observation of brainstem activation with MEG is very difficult. It would be interesting to compare our results to PET or fMRI data investigating swallowing in ALS patients

### Shift of cortical lateralization

The second main finding of this study was a disease-related shift of hemispheric lateralization of swallowing. While healthy subjects showed a slightly stronger left hemispheric activation, the right sensorimotor cortex was predominantly involved in ALS patients. Interestingly, this effect seems to increase with disease progression.

The question of lateralization can be interpreted in two ways: either by an increased activation of the right hemisphere or by a reduction of the left one. In principle it would be possible to interpret our findings as a greater pathological loss of cortical activation at the left as compared to the right sensorimotor cortex. However, anatomical and morphological studies on ALS patients with and without dysphagia did not demonstrate predominance of left hemispheric involvement [23]. Additionally, this hypothesis has no clear link to previous studies on human swallowing and does not carry a meaningful hypothesis with regard to cortical compensation of dysphagia in ALS. Therefore, we believe that the effect can be explained by an increase of right hemispheric activation. This phenomenon can be explained by the hypothesis of a task sharing for different components of deglutition between the two hemispheres already mentioned in the introduction section, saying that the left hemisphere predominantly processes the oral phase and the right hemisphere the pharyngeal phase of deglutition. This was hypothesized by Daniels and co-workers after a dual-task paradigm was used to examine deglutition [33] and underlined by a previous MEG study of our group[10]. Here we could show that the early stage of swallowing is associated with distinct left hemispheric cortical activation followed by a shift to the right hemisphere in the later stage of deglutition. The influence of right hemispheric stroke on the pharyngeal transit duration and a prolonged oral transit time after left hemispheric stroke were already demonstrated about 13 years earlier by Robbins and co-workers [15]. Similarly, another study showed that right hemispheric stroke led to dysfunction and dysmotility of the pharyngeal stage of deglutition [34].

In the present study ALS patients showed pharyngeal phase dysphagia, resulting in residues in the valleculae and sinus pyriformes, a finding which was also described by Leder and co-workers [35]. In keeping with this pattern, disturbed pharyngeal bolus transport, weak pharyngeal constriction and stasis in the pyriform sinus had been identified as main pathological finding in ALS patients by Higo and co-workers using videofluoroscopy [36]. Interestingly, in that study additional disturbances of the oral phase appeared only later in the course of the disease. Finally, investigation of ALS-related dysphagia by EMG of submental und cricopharyngeal muscles also resulted in pharyngeal phase problems, in particular a delayed triggering of the swallowing reflex was observed [3]. All these results show that pharyngeal phase dysphagia is prominent in patients with ALS.

We therefore suppose that the right hemispheric lateralization of swallowing is the only and admittedly weak sign of cortical plasticity in dysphagic ALS patients observed in this study. By increasing the number of active neurons compared to the left hemisphere disordered pharyngeal swallowing is partly compensated. This hypothesis is corroborated by the previously mentioned study on patients with KD [9]. Similar to ALS and due to weakness of bulbar muscles, KD leads to pharyngeal phase dysphagia. Apart from an overall increase of sensorimotor cortex activation a right-hemispheric lateralization was observed in this patient collective.

### Limitations

Limitations of the present study are the blindness of the magnetoencephalography to subcortical activations mainly caused by the muscular swallowing artefact. Due to head movements during recordings and the lack of individual MRIs localization of activation is not very accurate. Furthermore a distinction of the patients in relation to the predominantly involved motor neuron was not possible. Most patients presented with a combination of bulbar and pseudobulbar symptoms causing dysphagia. Fibrillations of the tongue were observed in nearly all subjects in both groups. No difference regarding the specificity of bulbar signs was found between mildly and severely affected patients. Future studies on ALS patients with higher group size should be performed to focus on this topic. The MEG swallowing task is less demanding compared to the swallowing screening test. We suppose that therefore the swallowing performance is comparable in patients and healthy controls. Nevertheless, apart from the swallowing frequency and EMG power no further parameters were recorded. It is therefore possible, that the observed difference in cortical swallowing activation can also be caused by behavioral differences. To further tease this out a parametric scanning design would be necessary, with differing levels of swallowing tasks. By this a better correlation between behavior and cortical activity would be possible. Further studies are necessary to further examine this point.

## Materials and Methods

### Subjects

Fourteen patients were diagnosed with bulbar–onset ALS (5 female, 9 male, age-range 44–74 years, mean 58.9 years) in accordance with the revisited El-Escorial-criteria [37] (see table 4). ALS severity was assessed with the ALS functional rating scale (ALSFRS) that grades motor dysfunction with regards to 10 subitems on a scale from 4 (normal function) to 0 (unable to attempt the task) [38]. According to the swallowing item of the ALSFRS patients were divided into two groups. Patients with 3 points (early eating problems – occasional choking) were considered as mildly dysphagic (MDG; n = 7), while patients with a score of 2 (dietary consistency changes) or lower were classified as severely dysphagic group (SDG; n = 7) (see table 5).

**Table 4:** Patients and control subjects characteristics with onset of the disease, and other diseases.

Patient	Age	Sex/Hand	Time after onset In month	Comorbidity
Severely dysphagic group				
A1	54	F/R	9	Hypothyreosis
A2	44	F/R	3	Hypertension
A3	67	M/R	24	Obstructive apnea syndrome during sleep
A4	53	M/R	27	---
A5	59	F/M	6	Hypertension
A6	60	M/R	18	Depression
A7	54	M/R	12	---
	55.9 +/− 6.6	4 male	14.1 +/− 8.4	
Mildly dysphagic group				
B1	68	M/R	6	Diabetes
B2	65	M/R	9	St. p. cardiac infarction
B3	53	M/R	3	Polyneuropathy
B4	62	F/R	60	Struma nodosa, Lumbago, Depression
B5	74	M/R	7	Asthma bronchiale
B6	45	F/R	8	Lumbal herniated disk
B7	51	M/R	18	---
	59.7 +/− 9.6	5 male	15.9 +/− 18.5	
Healthy control group				
C1	71	F/R		Diabetes
C2	71	F/R		---
C3	56	M/R		Hypertension
C4	60	M/R		Hypertension
C5	47	M/R		---
C6	57	F/R		Hyperthyreosis
C7	41	M/R		---
	57.6 +/− 10.4	4 male		

**Table 5:** Amyotrophic Lateral Sclerosis Functional Rating Scale (ALSFRS); range 4 – 40; bulbar score  =  subscore speech + subscore swallowing; 1–10 in each subscore category.

Patients	ALSFRS	Selected subitems				
		Speech	Swallowing	Salivation	Handwriting	Walking
A1	33	2	2	1	4	4
A2	33	1	2	2	4	4
A3	24	2	2	2	2	3
A4	24	0	1	2	3	3
A5	32	3	2	2	3	4
A6	20	0	1	0	2	3
A7	27	3	2	3	2	3
Mean	27.6	1.6	1.7	1.7	2.9	3.4
B1	33	4	3	1	3	3
B2	37	3	3	3	4	4
B3	27	2	3	1	3	3
B4	36	3	3	4	4	4
B5	32	3	3	4	3	3
B6	31	3	3	3	3	3
B7	34	3	3	3	4	3
Mean	32.9	3	3	2.7	3.4	3.3

As is outlined in table 4 and table 5, SDG patients were slightly younger (55.9 vs. 62.1) and had a smaller mean ALSFRS score than MDG patients. The mean swallowing subscore was 1.7 in SDG and 3 in MDG patients (p < 0.05). In both groups all patients presented with signs of the upper (pyramidal signs including exaggerated reflexes and spasticity) and lower motoneuron (fasciculation, paresis). Regarding bulbar symptoms, five patients in the MDG and four patients in the SDG presented with definite symptoms of the lower motoneuron including fasciculations, tongue atrophy or tongue fibrillation. Two patients additionally presented with pseudobulbar affect. Therefore dysphagia must be considered as a combination of bulbar and pseudobulbar palsy.

Seven healthy subjects without any history of neurological or ear-nose-throat-disorders served as controls (3 female, 4 male, age-range 41–71, mean 57.6 years).

All participants underwent clinical examination and MEG swallowing measurement. Before MEG recording was started a dysphagia screening test was performed according to the protocol by Hughes and Wiles (1996). Each subject drank 150 ml of water from a plastic beaker. They were instructed to drink ‘as quickly and as comfortably possible’. Subjects were observed from the side, and the number of swallows counted by observing the movements of the thyroid cartilage. A stopwatch was started when the water first touched the bottom lip, and stopped when the larynx came to rest for the last time [39]. ALS patients additionally underwent fiberoptic endoscopic evaluation of swallowing (FEES), based on the protocol published by Langmore and previously applied in our department [40]. FEES allows visualization of the pharyngeal swallow and by this the evaluation of penetration, aspiration, leakage and residues [41].

Only healthy subjects (healthy control subjects and ALS patients) or those suffering from minor comorbidities were included. Regarding diabetes the HbA1c was below 6.5% in all participants and diabetes was unexceptionally treated by diet or oral medication. Previous severe diabetic complications like cranial nerve palsies or metabolic decompensation had been excluded. Polyneuropathy was only associated with sensory symptoms like hypo- and dysesthesia of the feet, but no beyond this. Affection of the arm or cranial nerves was excluded by electrophysiological examination. Affection of the thyroid was not accompanied by an enlarged thyroid or any other signs of hypothyroidism.

The local ethics committee approved the protocol of the study. Informed consent was obtained from each subject after the nature of the study was explained, in accordance to the principles of the Declaration of Helsinki.

### MEG recording

The minimum variance beamformer is an approach for reconstruction of spatio-temporal brain activities from neuromagnetic measurements. As the MEG beamformer does not rely on averaging (across trials) to increase the signal-to-noise ratio, this method is capable of analyzing both evoked and induced brain activity [42]. An additional advantage of MEG is that subjects can be studied in a sitting position thereby avoiding any potential confounds caused by an experimental design requiring an unphysiological supine position during swallowing. Problematic are the artifacts caused by oropharyngeal muscle activation during deglutition, which make it difficult to study activation in subcortical and bulbar structures [8], [43]. However, cortical areas and especially sensorimotor areas can be examined in detail. In the last few years beamforming has been demonstrated to be a reliable method to examine complex sensorimotor functions [44], especially swallowing [7], [8], [9], [12], whereas motor tasks result in ERD of the beta-rhythm in cortical motor areas [5].

To facilitate swallowing during MEG recording water was infused into the oral cavity via a flexible plastic tube 4.7 mm in diameter attached to a fluid reservoir. The reservoir bag was positioned about 1 m above the mouth of each subject when seated. The tip of the tube was placed in the corner of the mouth between the buccal part of the teeth and the cheek. The tube was gently fixed to the skin with tape. The side chosen for tube placement was alternated between subjects. The infusion flow was individually adjusted to the subject's request and ranged between 8 and 12 ml/min. The aim was to establish a swallowing frequency of four to six times per minute. Additionally the swallowing task during MEG recordings had to be safe and without any risk of aspiration for all participating patients. For ethical reasons we have chosen an easy to perform paradigm with small volumes per swallow. During 15 minutes of MEG recording, the subject swallowed in a self-paced manner, without external cueing. Swallowing acts were recorded and identified by electromyographic recording. Surface EMG was measured with two pairs of bipolar skin electrodes (Ag-AgCl) placed on the submental muscle groups [45]. Muscles were palpated by a medical technician during deglutition prior to MEG recordings and the electrodes were adjusted to skin with tape stripes. The electrodes were connected to a bipolar amplifier (DSQ 2017E EOG/EMG system, CTF Systems Inc., Canada). EMG data was high pass filtered with 0.1Hz before markers were manually set. MEG data were collected using a whole head 275-channel SQUID sensor array (Omega 275, CTF Systems Inc., Canada) housed in a magnetically shielded room. Magnetic fields were recorded with a sample frequency of 600 Hz. The data were filtered during acquisition using a 150 Hz low-pass filter. Recordings were performed while subjects were seated in a comfortable upright position and watching a silent movie of their choice. During MEG recordings subjects and patients head movements were recorded. Only measurements with a total head movement of less than 1 cm were taken into account for further calculations. Furthermore subjects were observed on an online video screen during recordings by a medical technician to exclude unrequested task linked movements. Measurements with more than 1 cm head movement of observed unrequested movements were canceled and had to be repeated. This occurred in one patient of the SDG and in one control subject. No difference in the extent of head movement was observed between the groups. Furthermore, subjects were observed via an online video screen during recordings by a medical technician to exclude any other task-related movements.

### Data analysis

**Figure 2:**
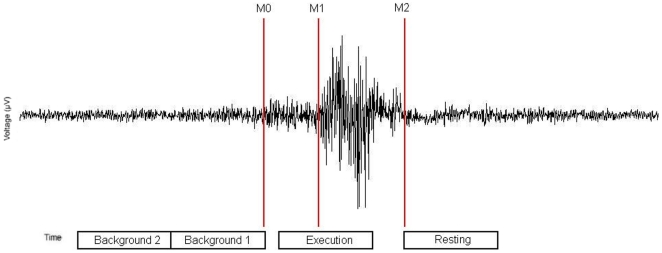
Definition of active and resting stage of swallowing-related muscle activity. The EMG recording of one swallowing act is shown (surface electrodes, recording from the submental muscles). For the analysis with SAM, the beginning (M_1_) and the end (M_2_) of main muscle activation were marked. The activation phase and the corresponding resting phase were defined. To estimate the maximal null distribution a third marker (M_0_) at the beginning of preparation activity was set and two background phases were defined (Methods).

Each individual's EMG signal was used to manually mark the beginning of main muscle activation (M_1_) and the end of the task-specific muscle activity (M_2_) for every single swallow. The examiner who set the markers to the datasets was blinded to the group affiliation of the subjects. Before marker setting a band pass filter 3–100 Hz was applied to data. The beginning of the main muscle activation was defined as an enduring >100% increase in amplitude or frequency of the EMG signal after an initial increase of more than 50% defining the onset of swallowing preparation (M_0_) (see figure 2). The end of task-specific muscle activity was defined as a decrease in amplitude/frequency of EMG signal >50%. In all subjects, the movement stage lasted longer than 1 s in more than 90% of the trials. Only these trials were taken into account. For further analysis time intervals were defined as following:

1. Execution stage: −.4 to .6 s in reference to M_1_
2. Resting stage: 0 to 1 s in reference to M_2_
3. Background stage 1: −1 to −0 s in reference to M_0_
4. Background stage 2: −2 to −1 s in reference to M_0_

About five percent of the trials were rejected due to overlap between (1) and (2); (1) and (3) or between (4) and (2) of the subsequent swallow in each group. By this a contamination of data by the post-movement phase and pre-movement activation could be avoided. The time intervals of (3) and (4) were used to estimate the maximum null distribution. According to previous SAM results on motor tasks, MEG data were filtered within the beta frequency range (13–30 Hz) and the two adjacent bands (alpha: 8–13 Hz; gamma: 30–60 Hz). From the filtered MEG data, SAM was used to generate a 20×20×14 cm volumetric pseudo-t images [46], with 3-mm voxel resolution for all three frequency bands. A pseudo-t value [46], [47] cancels the common-mode brain activity (‘brain noise’) by subtracting the source power found in a defined control stage from the source power in the active stage. To account for sensor noise, which is spuriously mapped to the brain as inhomogeneously distributed background activity, this difference is normalized by an estimation of the mapped noise power (sensor noise power converted to its locally varying contribution to the reconstructed source power). For analyzing cortical activity during the execution stage (1), the corresponding resting stage (2) served as control. The required similarity between the resting stage and the two background stages in patients as well as in controls was proven before by a direct comparison of these 3 stages. Therefore a standard permutation test for paired samples was performed on these time intervals and we assured that no significant activation is found in this direct comparison.

As no structural MRIs were available in the ALS patients normalization procedure was performed as previously published by our group [48]. Briefly, the individual SAM images were mapped on the MNI (Montreal Neurological Institute) space using the knowledge about the fiducial point positions in the MEG coordinate system. The MNI space is also used in SPM2, and corresponds approximately to the Talairach space. Then, the rotated SAM image was shifted so that the center of the spherical head model used in the SAM calculation coincided with the center of the head model calculated for the template. In a last step the size of the SAM image was adjusted so that the radii of the head models in both images matched. Compared to an established MRI based normalization procedure (SPM2), the new method shows only minor errors of about 0.5 cm in single subject results as well as in group analysis [48].

For SAM analysis of single conditions, the significance of activated brain regions was assessed by the permutation test method described by Chau and co-workers [49], [50]. The maximal null distribution was estimated by comparing Background stage 1 (active) and 2 (control) [49], [50]. For the comparison of the different groups, a standard permutation test for unpaired samples was performed [50] and corrected for multiple tests using a Bonferroni correction.

Hemispheric lateralization of brain activation was quantified using a lateralization index (LI), which was calculated as (L−R)/(L+R), where L and R are the cumulative pseudo-t values in the primary sensorimotor cortex (BA 3, 1, 2 and 4, according to the Talairach atlas) of the left and right hemispheres, respectively. A comparison of the pseudo t-values of both hemispheres is unproblematic due to the comparable noise the whole head system projects on both sides. A positive LI indicates left hemispheric lateralization, while a negative LI indicates stronger right hemispheric activation. Ratios around 0 represent indeterminate dominance, 1 or, −1 respectively, are indicating unilateral activation [7], [51].

## References

[1] Brooks BR (1996). Clinical epidemiology of amyotrophic lateral sclerosis.. Neurol Clin.

[2] Hillel A, Dray T, Miller R, Yorkston K, Konikow N (1999). Presentation of ALS to the otolaryngologist/head and neck surgeon: getting to the neurologist.. Neurology.

[3] Ertekin C, Aydogdu I, Yuceyar N, Kiylioglu N, Tarlaci S (2000). Pathophysiological mechanisms of oropharyngeal dysphagia in amyotrophic lateral sclerosis.. Brain.

[4] Hamalainen MS (1992). Magnetoencephalography: a tool for functional brain imaging.. Brain Topogr.

[5] Pfurtscheller G, Andrew C (1999). Event-Related changes of band power and coherence: methodology and interpretation.. J Clin Neurophysiol.

[6] Jurkiewicz MT, Gaetz WC, Bostan AC, Cheyne D (2006). Post-movement beta rebound is generated in motor cortex: Evidence from neuromagnetic recordings.. Neuroimage.

[7] Dziewas R, Soros P, Ishii R, Chau W, Henningsen H (2003). Neuroimaging evidence for cortical involvement in the preparation and in the act of swallowing.. Neuroimage.

[8] Furlong PL, Hobson AR, Aziz Q, Barnes GR, Singh KD (2004). Dissociating the spatio-temporal characteristics of cortical neuronal activity associated with human volitional swallowing in the healthy adult brain.. Neuroimage.

[9] Dziewas R, Teismann IK, Suntrup S, Schiffbauer H, Steinstraeter O (2009). Cortical compensation associated with dysphagia caused by selective degeneration of bulbar motor neurons.. Hum Brain Mapp.

[10] Teismann IK, Dziewas R, Steinstraeter O, Pantev C (2009). Time-dependent hemispheric shift of the cortical control of volitional swallowing.. Hum Brain Mapp.

[11] Teismann IK, Steinstraeter O, Schwindt W, Ringelstein EB, Pantev C (2010). Age-related changes in cortical swallowing processing.. Neurobiol Aging.

[12] Teismann IK, Steinstraeter O, Stoeckigt K, Suntrup S, Wollbrink A (2007). Functional oropharyngeal sensory disruption interferes with the cortical control of swallowing.. BMC Neurosci.

[13] Teismann IK, Steinstraeter O, Warnecke T, Zimmermann J, Ringelstein EB (2008). Cortical recovery of swallowing function in wound botulism.. BMC Neurol.

[14] Teismann IK, Steinstrater O, Warnecke T, Suntrup S, Ringelstein EB (2009). Tactile thermal oral stimulation increases the cortical representation of swallowing.. BMC Neurosci.

[15] Robbins J, Levine RL, Maser A, Rosenbek JC, Kempster GB (1993). Swallowing after unilateral stroke of the cerebral cortex.. Arch Phys Med Rehabil.

[16] Hamdy S, Rothwell JC, Brooks DJ, Bailey D, Aziz Q (1999). Identification of the cerebral loci processing human swallowing with H2(15)O PET activation.. J Neurophysiol.

[17] Hamdy S, Rothwell JC, Aziz Q, Singh KD, Thompson DG (1998). Long-term reorganization of human motor cortex driven by short-term sensory stimulation.. Nat Neurosci.

[18] Konrad C, Jansen A, Henningsen H, Sommer J, Turski PA (2006). Subcortical reorganization in amyotrophic lateral sclerosis.. Exp Brain Res.

[19] Kew JJ, Leigh PN, Playford ED, Passingham RE, Goldstein LH (1993). Cortical function in amyotrophic lateral sclerosis. A positron emission tomography study.. Brain.

[20] Kew JJ, Brooks DJ, Passingham RE, Rothwell JC, Frackowiak RS (1994). Cortical function in progressive lower motor neuron disorders and amyotrophic lateral sclerosis: a comparative PET study.. Neurology.

[21] Konrad C, Henningsen H, Bremer J, Mock B, Deppe M (2002). Pattern of cortical reorganization in amyotrophic lateral sclerosis: a functional magnetic resonance imaging study.. Exp Brain Res.

[22] Schoenfeld MA, Tempelmann C, Gaul C, Kuhnel GR, Duzel E (2005). Functional motor compensation in amyotrophic lateral sclerosis.. J Neurol.

[23] Li S, Chen Q, Yu B, Xue K, Luo C (2009). Structural and functional changes mapped in the brains of amyotrophic lateral sclerosis patients with/without dysphagia: a pilot study.. Amyotroph Lateral Scler.

[24] Mohammadi B, Kollewe K, Samii A, Krampfl K, Dengler R (2009). Decreased brain activation to tongue movements in amyotrophic lateral sclerosis with bulbar involvement but not Kennedy syndrome.. J Neurol.

[25] Smith CD (2002). Serial MRI findings in a case of primary lateral sclerosis.. Neurology.

[26] Kuipers-Upmeijer J, de Jager AE, Hew JM, Snoek JW, van Weerden TW (2001). Primary lateral sclerosis: clinical, neurophysiological, and magnetic resonance findings.. J Neurol Neurosurg Psychiatry.

[27] Le Forestier N, Maisonobe T, Spelle L, Lesort A, Salachas F (2001). Primary lateral sclerosis: further clarification.. J Neurol Sci.

[28] Butman JA, Floeter MK (2007). Decreased thickness of primary motor cortex in primary lateral sclerosis.. AJNR Am J Neuroradiol.

[29] Tanaka M, Ichiba T, Kondo S, Hirai S, Okamoto K (2003). Cerebral blood flow and oxygen metabolism in patients with progressive dementia and amyotrophic lateral sclerosis.. Neurol Res.

[30] Cosottini M, Giannelli M, Siciliano G, Lazzarotti G, Michelassi MC (2005). Diffusion-tensor MR imaging of corticospinal tract in amyotrophic lateral sclerosis and progressive muscular atrophy.. Radiology.

[31] Luis ML, Hormigo A, Mauricio C, Alves MM, Serrao R (1990). Magnetic resonance imaging in motor neuron disease.. J Neurol.

[32] Mosier KM, Liu WC, Maldjian JA, Shah R, Modi B (1999). Lateralization of cortical function in swallowing: a functional MR imaging study.. AJNR Am J Neuroradiol.

[33] Daniels SK, Corey DM, Fraychinaud A, DePolo A, Foundas AL (2006). Swallowing lateralization: the effects of modified dual-task interference.. Dysphagia.

[34] Daniels S, Foundas A, Iglesia G, Sullivan M (1996). Lesion site in unilateral stroke patients with dysphagia.. J Stroke Cerebrovasc Dis.

[35] Leder SB, Novella S, Patwa H (2004). Use of fiberoptic endoscopic evaluation of swallowing (FEES) in patients with amyotrophic lateral sclerosis.. Dysphagia.

[36] Higo R, Tayama N, Nito T (2004). Longitudinal analysis of progression of dysphagia in amyotrophic lateral sclerosis.. Auris Nasus Larynx.

[37] Brooks BR, Miller RG, Swash M, Munsat TL (2000). El Escorial revisited: revised criteria for the diagnosis of amyotrophic lateral sclerosis.. Amyotroph Lateral Scler Other Motor Neuron Disord.

[38] Hillel AD, Miller RM, Yorkston K, McDonald E, Norris FH (1989). Amyotrophic lateral sclerosis severity scale.. Neuroepidemiology.

[39] Hughes TA, Wiles CM (1996). Clinical measurement of swallowing in health and in neurogenic dysphagia.. Qjm.

[40] Dziewas R, Warnecke T, Schnabel M, Ritter M, Nabavi DG (2007). Neuroleptic-induced dysphagia: case report and literature review.. Dysphagia.

[41] Langmore SE, Schatz K, Olson N (1991). Endoscopic and videofluoroscopic evaluations of swallowing and aspiration.. Ann Otol Rhinol Laryngol.

[42] Robinson S, Vrba J, Yoshimoto T, Kotani M, Kuruki S, Karibe H, Nakasato N (1999). Functional neuroimaging by synthetic aperture magneometry (SAM)..

[43] Robbins J, Levin RL (1988). Swallowing after unilateral stroke of the cerebral cortex: preliminary experience.. Dysphagia.

[44] Taniguchi M, Kato A, Fujita N, Hirata M, Tanaka H (2000). Movement-related desynchronization of the cerebral cortex studied with spatially filtered magnetoencephalography.. Neuroimage.

[45] Vaiman M, Eviatar E, Segal S (2004). Surface electromyographic studies of swallowing in normal subjects: a review of 440 adults. Report 1. Quantitative data: timing measures.. Otolaryngol Head Neck Surg.

[46] Vrba J, Robinson SE (2001). Signal processing in magnetoencephalography.. Methods.

[47] Sekihara K, Sahani M, Nagarajan SS (2005). Localization bias and spatial resolution of adaptive and non-adaptive spatial filters for MEG source reconstruction.. Neuroimage.

[48] Steinstraeter O, Teismann IK, Wollbrink A, Suntrup S, Stoeckigt K (2009). Local sphere-based co-registration for SAM group analysis in subjects without individual MRI.. Exp Brain Res.

[49] Chau W, McIntosh AR, Robinson SE, Schulz M, Pantev C (2004). Improving permutation test power for group analysis of spatially filtered MEG data.. Neuroimage.

[50] Nichols TE, Holmes AP (2002). Nonparametric permutation tests for functional neuroimaging: a primer with examples.. Hum Brain Mapp.

[51] Yetkin FZ, Hammeke TA, Swanson SJ, Morris GL, Mueller WM (1995). A comparison of functional MR activation patterns during silent and audible language tasks.. AJNR Am J Neuroradiol.

